# Dose–Response Association Between Handgrip Strength and All‐Cause Mortality Across Different Levels of Systemic Inflammation

**DOI:** 10.1002/jcsm.70272

**Published:** 2026-03-27

**Authors:** Andrea Tur‐Boned, Lars Louis Andersen, Rubén López‐Bueno, Rodrigo Núñez‐Cortés, Carlos Cruz‐Montecinos, Luis Suso‐Martí, Ana Polo‐López, Joaquín Calatayud

**Affiliations:** ^1^ Exercise Intervention for Health Research Group (EXINH‐RG), Department of Physiotherapy University of Valencia Spain; ^2^ National Research Centre for the Working Environment Copenhagen Denmark; ^3^ Department of Health Sciences Public University of Navarra Pamplona Spain; ^4^ Department of Physical Therapy, Faculty of Medicine University of Chile Santiago Chile

**Keywords:** C‐reactive protein, dynamometer, muscle strength, older adults, prospective

## Abstract

**Background:**

Longitudinal associations between C‐reactive protein (CRP) and handgrip strength from cohort studies remain inconsistent. We investigated the dose–response association between handgrip strength and all‐cause mortality across different levels of systemic inflammation.

**Methods:**

We analysed data from 20 941 participants (mean age 68 years ±9.4 SD; 43.1% men) from the Survey of Health, Ageing and Retirement in Europe (SHARE) Waves 6 to 9. CRP levels were categorized as > 3, > 10 and > 25 mg/L. Time‐varying Cox proportional hazards regression with restricted cubic splines modeled associations between handgrip strength and all‐cause mortality, adjusting for age, sex, BMI, smoking, education, marital status, geographic region and medications.

**Results:**

During 4.8–5.3 years follow‐up, mortalities were 14.7% (CRP > 3 mg/L), 22.5% (CRP > 10 mg/L) and 30.2% (CRP > 25 mg/L). A curvilinear dose–response association existed between handgrip strength and mortality across all CRP levels with a significant interaction found between CRP and handgrip strength (*p* = 0.0131). Using median handgrip strength as reference (30 kg for CRP > 3 mg/L; 28 kg for CRP > 10 and > 25 mg/L), participants at the 10th percentile showed hazard ratios of 1.84 (95% CI 1.59–2.14), 1.69 (95% CI 1.33–2.15) and 1.92 (95% CI 1.17–3.17), respectively. Participants at the 90th percentile demonstrated protective effects: HR 0.57 (95% CI 0.43–0.74), 0.50 (95% CI 0.30–0.82) and 0.38 (95% CI 0.17–0.87).

**Conclusions:**

Handgrip strength demonstrates a strong inverse dose–response association with mortality across varying inflammation levels. Protective effects persist even with elevated inflammatory markers, supporting handgrip strength as an accessible biomarker for mortality risk stratification in older adults regardless of inflammatory status.

**Abbreviations**
BMI: body mass index
CI: confidence interval
CRP: C‐reactive protein
HR: hazard ratio
SHARE: Survey of Health, Ageing and Retirement in Europe
STROBE: Strengthening the Reporting of Observational Studies in Epidemiology

## 1 Introduction

C‐reactive protein (CRP), an acute‐phase reactant synthesized in the liver, exhibits elevated serum concentrations in response to systemic inflammation [1]. CRP is widely recognized as one of the foremost inflammatory biomarkers, being associated with loss of appendicular skeletal muscle and various physical and mental health disorders [2, 3, 4]. In fact, a meta‐analysis of cohort studies with 484 821 overall participants showed a curvilinear association between CRP and all‐cause and cardiovascular mortality and a linear association for non‐cardiovascular and cancer mortality [5].

Given that physical inactivity is also associated with elevated risks of age‐related diseases and mortality, low physical activity levels have been directly linked to increased anabolic resistance [6], higher CRP concentrations and elevated proinflammatory cytokine levels [7]. These findings underscore the critical role of regular exercise in preventing these physiological derangements, as it is found to be a protective tool against low grade systemic inflammation [8]. This long‐term effect of exercise may be attributed to the anti‐inflammatory response triggered by an acute bout of exercise, a process partially mediated by myokine signalling [8]. Notably, muscle contraction stimulates the production of anti‐inflammatory cytokines, further contributing to this protective mechanism [9]. Beyond these anti‐inflammatory effects, a key benefit of sustained physical activity and maintained fitness is the preservation or improvement of muscular strength parameters. Chronic low‐grade inflammation increases with aging [10], which has been associated with several deleterious health outcomes and may aggravate the phenomena of sarcopenia and dynapenia [11]. As muscle strength is a highly modifiable parameter in response to training interventions, handgrip strength has been established as a reliable biomarker for both overall muscular strength [12] and mortality [13]. Cross‐sectional studies have consistently reported an inverse association between systemic inflammation and handgrip strength across various populations, including men [14], women [15] and both genders in older adults [16]. However, a critical translational gap persists, as longitudinal and prognostic evidence remains scarce and inconsistent. For instance, a cohort study conducted among 2705 English older adults with 8‐year follow‐up found an inverse association between handgrip strength and change in inflammatory markers, albeit evident only in women [17].

Consequently, despite established links between inflammation, strength and mortality, current clinical practice lacks a crucial evidence base; no large‐scale prospective study has determined if handgrip strength predicts mortality differentially across inflammatory states. This gap hinders the development of nuanced, inflammation‐informed assessment protocols and represents a significant missed opportunity for risk stratification in the millions of older adults worldwide with elevated inflammatory markers. Without evidence‐based handgrip strength thresholds stratified by inflammatory status, clinicians cannot reliably use this simple, cost‐effective measure to identify high‐risk patients who would benefit most from early interventions. Given the aging global population and rising healthcare costs, establishing these dose–response associations is urgently needed to transform handgrip strength into a practical, validated biomarker that can guide clinical decision‐making and resource allocation in primary care and community settings.

The aim of this study was to examine the dose–response association of handgrip strength with all‐cause mortality in a representative sample of European older adults with different elevated levels of systemic inflammation measured with CRP concentrations.

## 2 Methods

### 2.1 Participants and Design

This study uses data from Waves 6–9 of the Survey of Health, Ageing and Retirement in Europe (SHARE) study, which includes 28 countries (27 European countries and Israel) [18]. Data were collected from February 2015 to December 2022. SHARE uses a multistage stratified sampling design in which involved countries are divided into different strata in relation to geographical area, and municipalities or postcodes within these strata served as primary sampling units. Data in each SHARE wave are collected through home computer‐assisted personal interviews. SHARE uses ex‐ante harmonized interviews, and new respondents are added in each wave to compensate for losses. The SHARE target population consists of all persons aged 50 years and over at the time of sampling who have their regular domicile in the respective SHARE country. In SHARE, persons are excluded from baseline or refreshment samples if they are incarcerated, hospitalized or out of the country during the entire survey period, unable to speak the country's language(s) or have moved to an unknown address. Partners living in the same household are interviewed regardless of their age. This study was reported according to the Strengthening the Reporting of Observational Studies in Epidemiology (STROBE) statement (Data S1).

In the present analyses, we included only individuals with CRP assessment collected in Wave 6 among 12 countries (Belgium, Denmark, Estonia, France, Germany, Greece, Italy, Slovenia, Spain, Sweden, Switzerland and Israel) participating in the blood biomarker evaluation and followed‐up during Waves 7,8 and/or 9. Among these 12 countries, there were 56 762 survey participants of whom 52 374 completed the survey interviews and 39 483 were eligible for the biomarker study. Respondents were ineligible for dried blood spot sampling if they had medical conditions preventing blood collection (such as bleeding disorders), were interviewed via proxy, were not part of a panel household in participating countries, lived outside the four eligible districts in France or were unable to provide consent themselves. Among the eligible participants, 27 373 consented (69%). In 2015, dried blood spot samples were collected from 27 200 of the 27 373 consenters. Among these, the final sample consisted of 20 941 individuals with valid CRP measurements and complete covariate data.

### 2.2 Dried Blood Spot Sample Collection and Processing

Dried blood spot samples were collected in Wave 6 by trained interviewers using a standardized kit and protocol. Blood was obtained via finger prick and dropped onto filter paper cards with preprinted circles. Cards were allowed to dry for at least 15 min before being sealed in plastic bags with desiccant and mailed to a central biobank at the University of Southern Denmark in Odense, Denmark. At the biobank, the samples were checked for quality and completeness before being stored at −20°C. Subsequently, the samples were punched and analysed in two batches (2017–2018 and 2020–2021) at the Department of Laboratory Medicine and Pathology, University of Washington in Seattle, USA.

The dried blood spot sampling methodology employed in SHARE Wave 6 was implemented under a rigorously standardized protocol to ensure both measurement validity and reproducibility. Validity was established through: (1) uniform collection materials and centralized interviewer training; (2) photometric spot‐size correction to account for blood volume variation; (3) batch analysis using calibrators and internal quality controls; and (4) statistical normalization of raw values, including conversion to venous blood equivalents via externally validated adjustment factors. Reproducibility (test–retest reliability) is supported by: the selection of biomarkers with previously demonstrated stability in dried blood spot matrices; stringent preanalytical procedures designed to minimize technical variability; and routine assessment of analytical precision (within‐ and between‐batch coefficients of variation) during laboratory processing. While the methodological report does not present study‐specific test–retest correlation coefficients, the protocol is explicitly grounded in prior evidence confirming strong correlations between dried blood spot‐derived and venous measurements for the included biomarkers, thereby ensuring the comparability and reliability of the obtained values. Full information regarding the SHARE Wave 6 methods including blood tests can be found elsewhere [19].

### 2.3 Exposure: Handgrip Strength

Maximal handgrip strength (kg) was evaluated using a calibrated Smedley‐type hand dynamometer (TTM, Tokyo, 100 kg capacity). The grip span was standardized on an individual basis by adjusting the dynamometer's inner lever to fit each participant's hand, specifically positioning it against the second phalanges to ensure an anatomically appropriate grip. Measurements were taken with participants preferentially standing, maintaining the upper arm against the trunk, elbow at a 90° angle and wrist in neutral position, although sitting was permitted if standing was not feasible. Participants were instructed to exert maximal force for 2 s. Standardized verbal encouragement was provided by trained assessors. Two trials were performed for each hand and the highest value recorded from either hand was used for analysis.

While an absolute test–retest reliability coefficient was not calculated for this specific dataset, the protocol's high consistency is evidenced by previous data, which showed extremely high correlations (*r* > 0.9) between repeated trials [20].

### 2.4 Outcome: All‐Cause Mortality

Mortality status and date of death were ascertained through proxy‐reported end‐of‐life interviews. Proxies, who were close contacts of the deceased (e.g., relatives, household members or neighbours), provided information on the date and cause of death. For cases where the precise date of death was unavailable, it was imputed as the midpoint between the date of the participant's last study interview and the date of the proxy interview.

### 2.5 Covariates

Analyses were controlled for age, sex, smoking, BMI, marital status, education, geographical region and number of medications. The interviewer noted the sex of the respondent based on observation and asked the respondent in case of uncertainty. Age was calculated as date of interview minus self‐reported date of birth. Smoking status was based on two questions about whether the respondent had ever smoked and was currently smoking, and it was categorized into one variable (current smoker, ex‐smoker and never smoked). BMI was calculated from self‐reported weight and height of the respondent (kg/m^2^). Respondents also replied to a question with six categories about marital status that was combined into four categories: (1) Married and living together with spouse or in a registered partnership, (2) divorced or married, but living separated from spouse, (3) never married and (4) widowed. Education was based on the question ‘What is the highest school leaving certificate or school degree that you have obtained?’ with response categories recoded into three ISCED‐1997 categories (low, medium and high education) (Eurostat 2024). Country was recoded into a new variable, geographical region, with five categories (Eastern, Western, Southern and Northern Europe and Israel) according to the United Nations definition (United Nations 2024). Respondents also replied to the question ‘Do you currently take drugs at least once a week for problems mentioned on this card?’ with 14 specific drugs for different health problems as well as an ‘other’ category and a ‘none’ category. A new categorical variable counting the number of drugs was constructed.

### 2.6 Statistical Analysis

We used time‐varying Cox proportional hazards regression to assess the association between handgrip strength and mortality risk. All covariates were treated as time‐varying in our model. To account for potential non‐linearity, we employed restricted cubic splines with knots at the 10th, 50th and 90th percentiles of the handgrip strength distribution.

We categorized CRP levels using the following cut‐points according to a previous study [21]: CRP > 3 mg/L (Low‐moderate risk); CRP > 10 mg/L (high risk) and CRP > 25 mg/L (very high risk). To visualize the association between handgrip strength and mortality risk, we plotted the estimated hazard ratios (HRs), using reference values of 30 kg for individuals with CRP > 3 mg/L and 28 kg for individuals with CRP > 10 mg/L and CRP > 25 mg/L. The median provides a robust central measure that anchors our restricted cubic spline models and allows for clear interpretation of mortality risk relative to the typical participant in each respective group. Results are presented as HRs for all‐cause mortality with 95% confidence intervals (CI). All analyses were adjusted for the aforementioned covariates.

All statistical analyses were performed using SAS version 9.4 (SAS Institute Inc., Cary, NC, USA). A *p*‐value less than 0.05 was considered statistically significant.

## 3 Results

**FIGURE 1:**
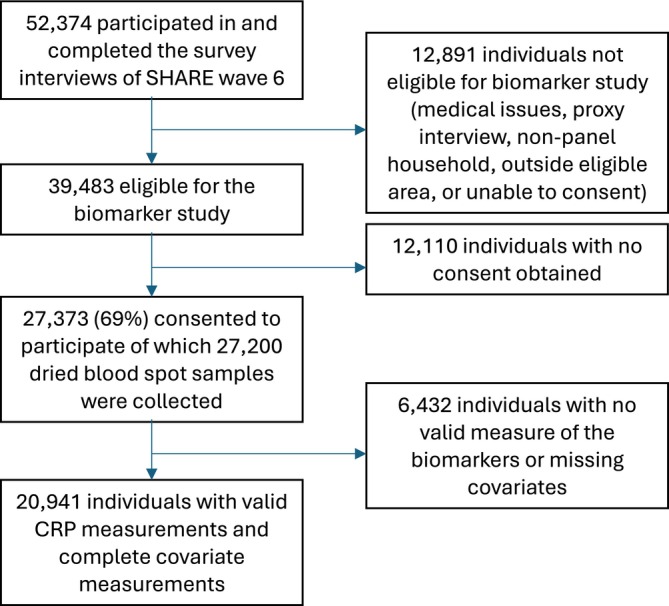
Flow of participants through the study.

The sample included 9020 (43.1%) men and 11 921 (56.9%) women with a mean age of 68 (SD 9.4) years, a mean CRP concentration of 3.5 (SD 6.6) mg/L and a mean handgrip strength of 33.3 (SD 11.8) kg. Figure 1 shows the flow of participants through the study. Table 1 shows the baseline characteristics of the study population of participants with different CRP levels (higher than 3, 10 and 25 mg/L). Handgrip strength levels were higher when CRP values were lower (CRP > 3 mg/L: 31.7 kg; CRP > 10 mg/L: 30.2 kg; CRP > 25 mg/L: 29.6 kg). A significant interaction was found between CRP and handgrip strength (*p* = 0.0131).

**TABLE 1:** Descriptive baseline characteristics of the study population.

	All regardless of CRP				CRP higher than 3				CRP higher than 10				CRP higher than 25			
	N	Mean	SD	Freq (%)	N	Mean	SD	Freq (%)	N	Mean	SD	Freq (%)	N	Mean	SD	Freq (%)
Handgrip strength (kg)	20 941	33.3	11.8		5932	31.7	11.6		854	30.2	11.6		252	29.6	11.4	
CRP_x4LFFB	20 941	3.5	6.6		5932	7.9	11.3		854	26.8	21.2		252	51.9	23.4	
Age	20 941	68	9.4		5932	68.7	9.5		854	69.6	9.5		252	71.4	9.4	
Body mass index (BMI)	20 941	27	4.7		5932	28.7	5.3		854	29	6.4		252	27.8	6.2	
Sex																
Male	9020			43.1	2436			41.1	363			42.5	123			48.8
Female	11 921			56.9	3496			58.9	491			57.5	129			51.2
Smoking status																
Smoker	3635			17.4	1203			20.3	202			23.6	51			20.2
Ex‐smoker	6446			30.8	1803			30.4	255			29.9	79			31.4
Never smoked	10 860			51.8	2926			49.3	397			46.5	122			48.4
Education (ISCED‐1997)																
Lower	7066			33.8	2356			39.7	338			39.6	99			39.3
Medium	8383			40	2292			38.6	335			39.2	98			38.9
Higher	5492			26.2	1284			21.7	181			21.2	55			21.8
Marital status																
Married and living togther with spouse or in a registered partnership	14 552			69.5	3996			67.4	548			64.2	162			64.3
Divorced or married but living separated from spouse	2097			10	617			10.4	85			9.9	17			6.7
Never married	1206			5.8	357			6	56			6.6	14			5.6
Widowed	3086			14.7	962			16.2	165			19.3	59			23.4
Geographic region (United Nations definition)																
Northern Europe	7877			37.6	2129			35.9	320			37.5	97			38.5
Southern Europe	5155			24.6	1608			27.1	208			24.3	63			25
Western Europe	7173			34.3	1938			32.7	287			33.6	92			36.5
Israel	736			3.5	257			4.3	39			4.6				
Number of medications																
0	4642			22.2	938			15.8	105			12.3	29			11.5
1	5443			26	1442			24.3	191			22.4	52			20.6
2	4269			20.4	1308			22.1	180			21.1	59			23.4
3	2985			14.2	907			15.3	137			16	37			14.7
4	1800			8.6	622			10.5	112			13.1	34			13.5
5	1802			8.6	715			12	129			15.1	41			16.3

Abbreviations: CRP = C‐reactive protein; SD = standard deviation.

When higher CRP values, increased number of deaths were reported over the follow‐up period. On the group of CRP > 3 mg/L, a total of 872 participants died (14.7%) over a follow‐up time of 5.3 years, while the group of CRP > 10 mg/L reported 192 deaths (22.5%) during a follow‐up period time of 5.1 years. Moreover, over 4.8 years of follow up, 76 deaths (30.2%) were reported in the group with CRP > 25 mg/L.

**FIGURE 2:**
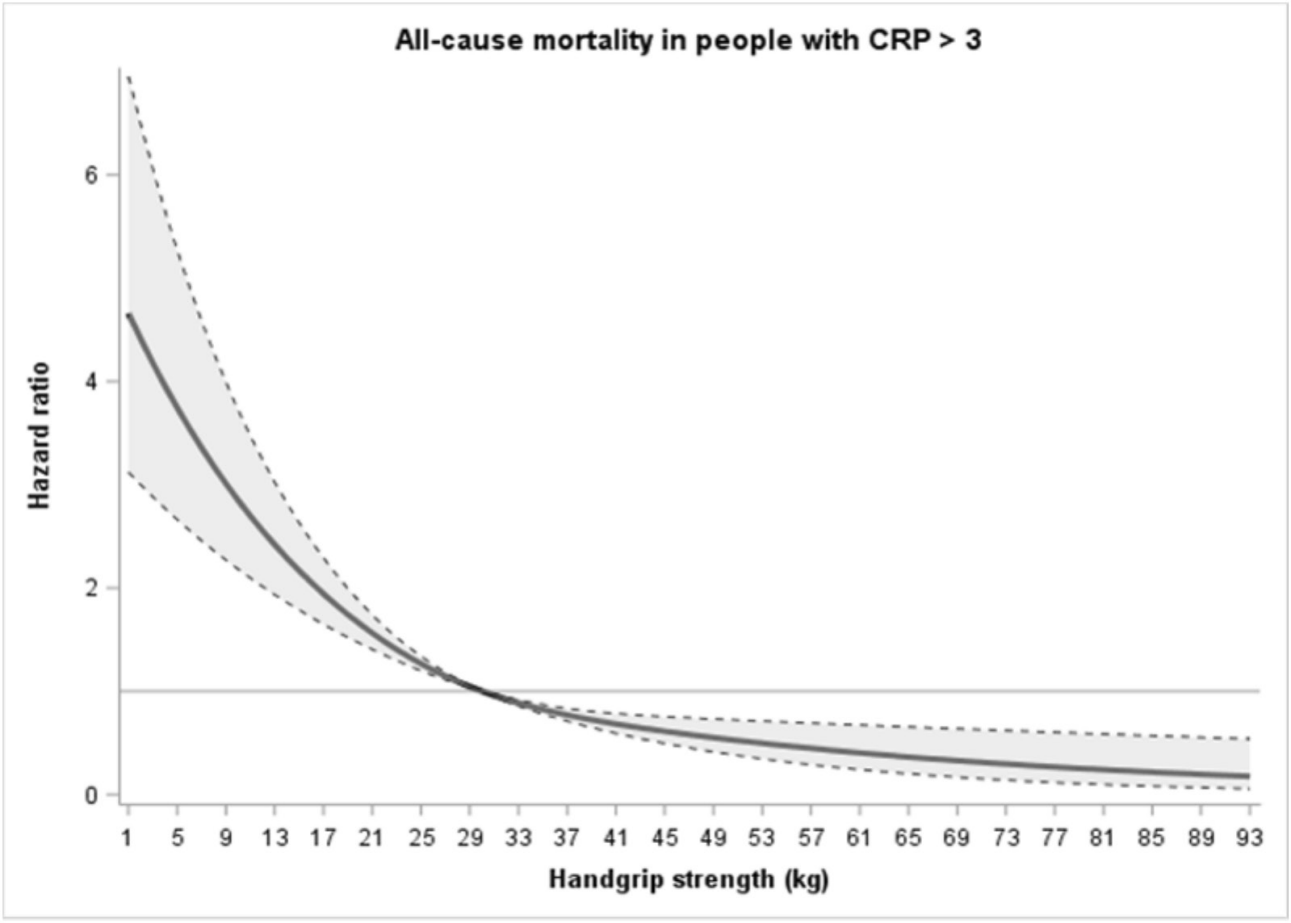
Dose response associations (Adjusted hazard ratios and associated 95% confidence interval band) between handgrip strength (kg) and all‐cause mortality in participants with CRP > 3 mg/L. Model adjusted for age, sex, body mass index (BMI), smoking status, education level, marital status, geographical region and the number of medications. Reference 30 kg (median handgrip strength). The continuous line represents hazard ratio values, whereas dotted lines represent 95% CI.

Figure 2 shows the grad**e**d association of handgrip strength with mortality risk during follow‐up in a sample of participants with CRP > 3 mg/L. Using the median level of muscle strength as reference (30 kg), lower and higher levels were associated in a curvilinear fashion with higher and lower mortality risk, respectively. The 10th percentile of muscle strength (18 kg) showed a HR of 1.84 (95% CI 1.59–2.14), while the 90th percentile (48 kg) showed a HR of 0.57 (95% CI 0.43–0.74).

**FIGURE 3:**
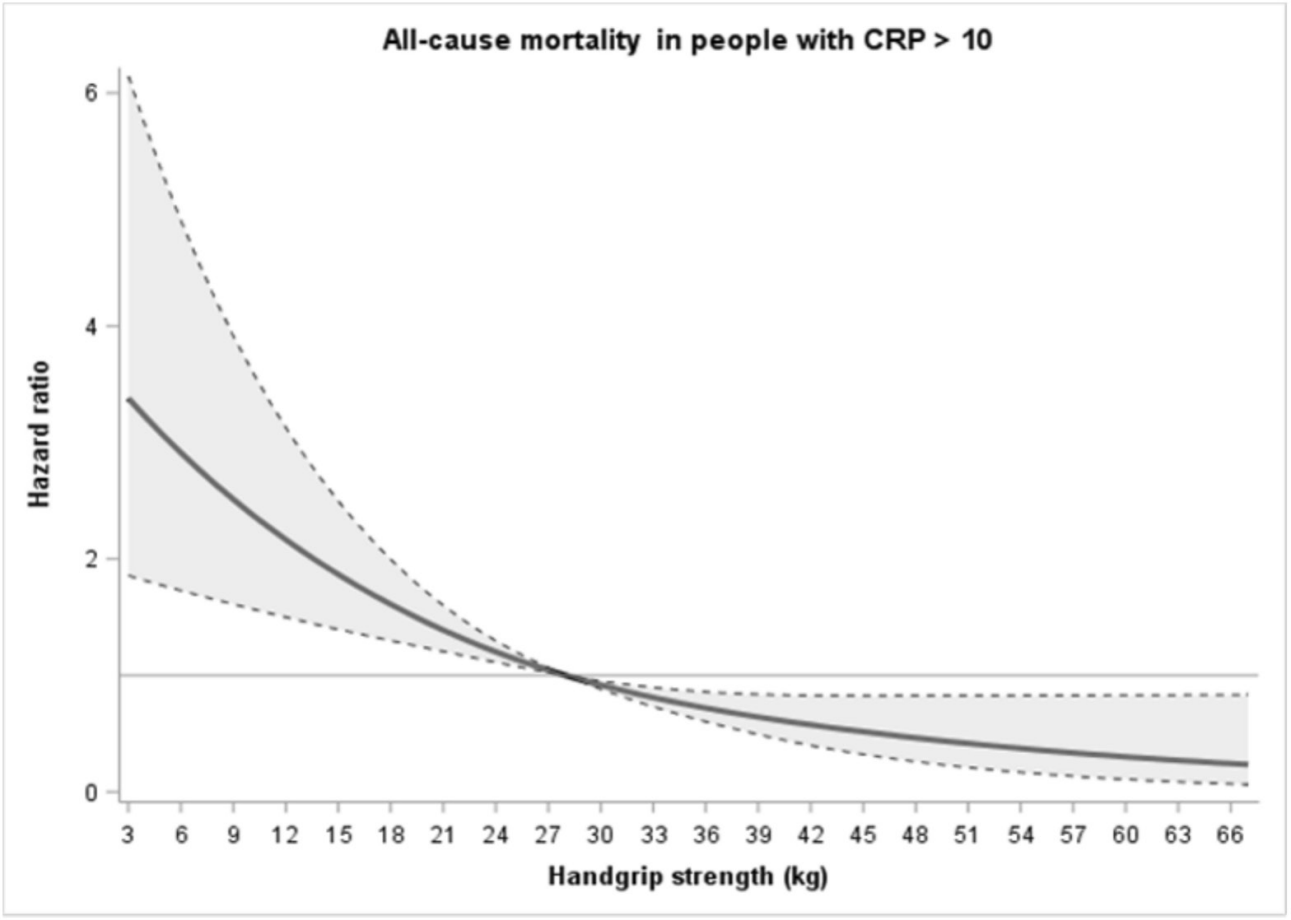
Dose response associations (Adjusted hazard ratios and associated 95% confidence interval band) between handgrip strength (kg) and all‐cause mortality in participants with CRP > 10 mg/L. Model adjusted for age, sex, body mass index (BMI), smoking status, education level, marital status, geographical region and the number of medications. Reference 28 kg (median handgrip strength). The continuous line represents hazard ratio values, whereas dotted lines represent 95% CI.

Figure 3 shows the graded association of handgrip strength with mortality risk during follow‐up in a sample of participants with CRP > 10 mg/L. Using the median level of muscle strength as reference (28 kg), lower and higher levels were associated in a curvilinear fashion with higher and lower mortality risk, respectively. The 10th percentile of muscle strength (17 kg) showed a HR of 1.69 (95% CI 1.33–2.15), while the 90th percentile (46 kg) showed a HR of 0.50 (95% CI 0.30–0.82).

**FIGURE 4:**
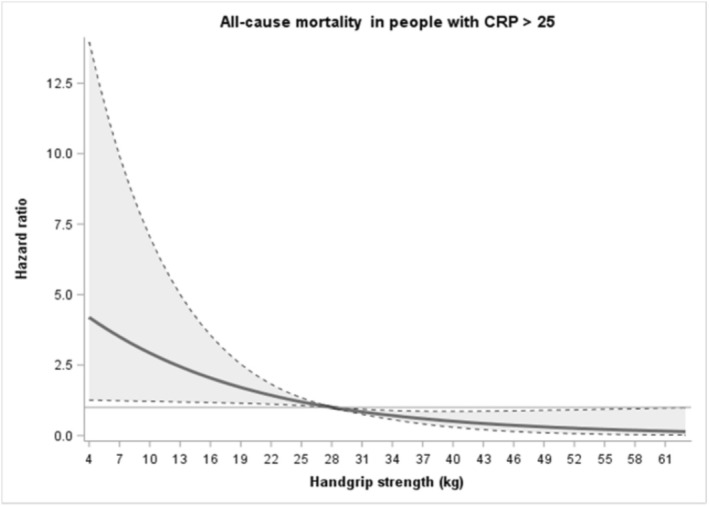
Dose response associations (Adjusted hazard ratios and associated 95% confidence interval band) between handgrip strength (kg) and all‐cause mortality in participants with CRP > 25 mg/L.Model adjusted for age, sex, body mass index (BMI), smoking status, education level, marital status, geographical region and the number of medications. Reference 28 kg (median handgrip strength). The continuous line represents hazard ratio values, whereas dotted lines represent 95% CI.

Figure 4 shows the graded association of handgrip strength with mortality risk during follow‐up in a sample of participants with CRP > 25 mg/L. Using the median level of muscle strength as reference (28 kg), lower and higher levels were associated in a curvilinear fashion with higher and lower mortality risk, respectively. The 10th percentile of muscle strength (17 kg) showed a HR of 1.92 (95% CI 1.17–3.17), while the 90th percentile (45 kg) showed a HR of 0.38 (95% CI 0.17–0.87).

## 4 Discussion

We investigated the dose–response association between handgrip strength and all‐cause mortality in adults with varying degrees of systemic inflammation (assessed via CRP levels) across European populations and Israel. A non‐linear association was observed: lower handgrip strength values were associated with increased mortality risk, whereas higher values correlated with reduced risk. Using median handgrip strength as the reference (30 kg for CRP > 3 mg/L, 28 kg for CRP > 10 mg/L and 28 kg for CRP > 25 mg/L), the analysis revealed a curvilinear trend, with mortality risk escalating progressively below the median and declining above it. Notably, our results demonstrate for the first time that handgrip strength provides differential, incremental prognostic value at distinct levels of inflammation, which is essential to move beyond a one‐size‐fits‐all approach. Such evidence could empower the development of targeted risk assessment protocols, allowing clinicians to utilize this low‐cost measure to identify high‐risk individuals, inform clinical decisions and optimize resource allocation in primary and community care.

Our findings, together with prior evidence, support the role of handgrip strength as a unifying clinical vital sign for systemic health and mortality risk prevention. Handgrip strength is consistently associated with all‐cause and cause‐specific mortality [13] and is widely used as an integrated marker of muscle function, muscle mass [12] and nutritional status [22]. Low handgrip strength is also linked to the incidence of multiple chronic conditions—ranging from cardiovascular [23], mental [24] and neurodegenerative diseases [25] to site‐specific cancers [26]. In this context, handgrip strength may capture the cumulative effect of lifelong health‐related behaviours associated with lower inflammatory profiles and other risk factors and, consequently, lower morbidity and mortality. This further suggests that interventions aimed at improving muscle mass and strength could be a strategic target for mitigating mortality among people with systemic inflammation.

Our most novel finding shows that handgrip strength reduced mortality risk even when high CRP was present. Unfotunately, to our knowledge, no other previous study evaluated associations between handgrip strength and mortality in people with different inflammation levels. However, the present results partially align with a previous eight‐year cohort study of 2705 adults reporting that higher baseline handgrip strength was separately associated with mortality and also with lower CRP levels, albeit only in females [17]. This negative association between handgrip strength and CRP has also been corroborated in another prospective cohort study among hospitalized patients [27] and additional cross‐sectional studies. For instance, a previous study conducted among women reported that a unit increase in absolute handgrip strength decreased the high‐sensitivity CRP level by 0.02 mg/dL [28]. In a study of 2116 Korean older adults, handgrip strength decreased linearly with increasing high‐sensitivity CRP levels in men, who exhibited 74.2% higher CRP when presenting low muscle strength [14]. Similarly, among 1131 Chinese older adults, elevated CRP was associated with lower muscle strength and poorer physical performance in men [29]. Comparable findings have been reported in 4186 English older adults [15], 2171 Korean postmenopausal women [30] and 262 Finnish nonagenarians [31]. This cross‐sectional association has also been observed in community‐dwelling older adults from the United States [16] and China [32], as well as in hospitalized patients [33].

Higher levels of CRP have been linked to an increased number of deaths [34]. In line with this, a cohort conducted among 14 238 middle‐aged Brazilian adults found progressively increased risk of all‐cause mortality with rising high‐sensitivity CRP concentrations [35]. Moreover, in a sex‐stratified Korean population study, a non‐linear association between blood CRP concentrations and all‐cause mortality was observed in men, whereas a weaker linear association was evident among women from a rural cohort [36]. Notably, while the shape of the association may vary by sex and population, the overall link is well‐established, as evidenced by a meta‐analysis of cohort studies which found a positive association between CRP levels and all‐cause mortality, with a 74% increased risk when comparing high level CRP (> 3 mg/L) with low level concentrations (< 1 mg/L) [5]. Furthermore, a significant dose–response association was observed, with each 1 mg/L rise in CRP associated with a 15% greater risk of all‐cause mortality [5].

Acting as an endocrine organ, skeletal muscle produces and releases cytokines and other small proteins—termed myokines—into the bloodstream. This release, especially during periods of muscle contraction, is capable of exerting systemic reductions in inflammation [9]. Among other inflammation markers changes after exercise, a small increase of CRP levels is seen the day after exercise of longer duration [37]. In this case, the role of CRP involves a dual mechanism as it facilitates the induction of anti‐inflammatory cytokines in circulating monocytes while concurrently suppressing the synthesis of proinflammatory cytokines in tissue macrophages [38]. This dual anti‐inflammatory role is supported by epidemiological evidence. For instance, physical activity was associated with significantly lower levels of inflammatory markers in a cross‐sectional study conducted among 5868 older adults in the United States [39]. Resistance training effectively reduces CRP concentrations and enhances functional capacity in healthy older adults [40]. Furthermore, longitudinal data strengthen this causal inference; the finding that regular exercise training induces a reduction in CRP levels [41, 42] suggests that physical activity itself can suppress systemic low‐grade inflammation. This is particularly salient for older populations, in whom baseline CRP levels are often elevated due to age‐related inflammatory processes [43], cardiovascular diseases [44] and various types of cancer [45] besides other ageing disorders, underscoring the potential of exercise to lessen a key driver of morbidity and mortality in this demographic.

### 4.1 Limitations

Our study has both strengths and limitations. We utilized a representative cohort of European adults with objectively measured handgrip strength and a critical inflammatory marker related to disease and mortality [34]. Our analysis accounted for a comprehensive set of covariates and potential confounding factors, ensuring the robustness of the results. In addition, we incorporated time‐varying handgrip performance and time‐varying measurements of relevant covariates into our analysis, decreasing the likelihood of biased estimates and improving the potential for identifying causal associations, although inherent time‐varying confounding cannot be discarded. However, the dried blood spots for the CRP measurements were only collected in Wave 6 and could therefore not be included as time‐varying, which is a limitation. Second, the possibility of residual confounding cannot be entirely ruled out. In fact, the absence of objective physical activity data as a potential confounder could remain a limitation of this study. Third, since the outcome was assessed through a proxy relative, there remains a risk of some degree of misclassification bias. Fourth, we did not include a detailed assessment of dietary quality, which may influence both inflammation and muscular health. While we cannot exclude residual confounding from unmeasured nutritional factors, a major strength of our analysis was the adjustment for BMI—a strong, objective proxy for long‐term energy balance and adiposity‐related metabolic inflammation [46, 47].

### 4.2 Future Directions

Our study focused exclusively on CRP, and future studies should evaluate other inflammatory markers such as interleukin‐6, tumour necrosis factor‐alpha and fibrinogen. Although handgrip strength was assessed, other functional markers could also have a protective benefit in mortality, requiring exploration in future studies. Finally, although our study sample comprised individuals within a mid‐to‐old age range, it is plausible that the protective benefits of handgrip strength extend to younger populations, warranting further investigation.

In conclusion, in adults with different levels of inflammation measured with CRP, an association exists between handgrip strength and all‐cause mortality, with a reduced risk observed at values above 30, 28 and 28 kg in participants with CRP > 3 mg/L, CRP > 10 mg/L and CRP > 25 mg/L values, respectively. Our results could contribute to highlighting the importance of muscular strength and its capacity to reduce mortality risk in this population.

## Funding

The authors have nothing to report.

## Ethics Statement

The SHARE database adhered to the principles of the World Medical Association (Declaration of Helsinki, last revised at the 64th WMA Meeting held in Fortalezza/Brazil in October 2013) and has obtained the approval of the Ethics Committee of Research in Humans of the Max Planck Society.

## Conflicts of Interest

The authors declare no conflicts of interest.

## Supporting information


**Data S1:** STROBE Statement—Checklist of items that should be included in reports of cohort studies.
